# Mechanisms of Hypoxia-Induced Pulmonary Arterial Stiffening in Mice Revealed by a Functional Genetics Assay of Structural, Functional, and Transcriptomic Data

**DOI:** 10.3389/fphys.2021.726253

**Published:** 2021-09-14

**Authors:** Edward P. Manning, Abhay B. Ramachandra, Jonas C. Schupp, Cristina Cavinato, Micha Sam Brickman Raredon, Thomas Bärnthaler, Carlos Cosme, Inderjit Singh, George Tellides, Naftali Kaminski, Jay D. Humphrey

**Affiliations:** ^1^Pulmonary, Critical Care and Sleep Medicine, Yale School of Medicine, New Haven, CT, United States; ^2^VA Connecticut Healthcare System, West Haven, CT, United States; ^3^Department of Biomedical Engineering, Yale University, New Haven, CT, United States; ^4^Respiratory Medicine, Hannover Medical School, Hannover, Germany; ^5^Vascular Biology and Therapeutics Program, Yale University, New Haven, CT, United States; ^6^Department of Anesthesiology, Yale School of Medicine, New Haven, CT, United States; ^7^Division of Pharmacology, Otto Loewi Research Center, Medical University of Graz, Graz, Austria; ^8^Department of Surgery, Yale School of Medicine, New Haven, CT, United States

**Keywords:** pulmonary artery, hypoxia, mouse, vascular, remodeling

## Abstract

Hypoxia adversely affects the pulmonary circulation of mammals, including vasoconstriction leading to elevated pulmonary arterial pressures. The clinical importance of changes in the structure and function of the large, elastic pulmonary arteries is gaining increased attention, particularly regarding impact in multiple chronic cardiopulmonary conditions. We establish a multi-disciplinary workflow to understand better transcriptional, microstructural, and functional changes of the pulmonary artery in response to sustained hypoxia and how these changes inter-relate. We exposed adult male C57BL/6J mice to normoxic or hypoxic (FiO_2_ 10%) conditions. Excised pulmonary arteries were profiled transcriptionally using single cell RNA sequencing, imaged with multiphoton microscopy to determine microstructural features under *in vivo* relevant multiaxial loading, and phenotyped biomechanically to quantify associated changes in material stiffness and vasoactive capacity. Pulmonary arteries of hypoxic mice exhibited an increased material stiffness that was likely due to collagen remodeling rather than excessive deposition (fibrosis), a change in smooth muscle cell phenotype reflected by decreased contractility and altered orientation aligning these cells in the same direction as the remodeled collagen fibers, endothelial proliferation likely representing endothelial-to-mesenchymal transitioning, and a network of cell-type specific transcriptomic changes that drove these changes. These many changes resulted in a system-level increase in pulmonary arterial pulse wave velocity, which may drive a positive feedback loop exacerbating all changes. These findings demonstrate the power of a multi-scale genetic-functional assay. They also highlight the need for systems-level analyses to determine which of the many changes are clinically significant and may be potential therapeutic targets.

## Introduction

The main pulmonary artery and associated first branch right and left pulmonary arteries are classified as elastic conduit vessels whose function is to facilitate blood flow from the right ventricle to the small pulmonary arteries, arterioles, and eventually capillaries where gas exchange occurs. This complex network of vessels regulates the ventilation:perfusion ratio by directing blood to regions of the lung where it can be best oxygenated. Medium-sized and small pulmonary arteries within the lung parenchyma can vasoconstrict to divert blood flow from alveoli that are not well-aerated or vasodilate to direct greater amounts of blood to alveoli that are well-aerated. The response of the pulmonary circulation to hypoxia is well-described in mammals. Hypoxia often results in acute and sustained narrowing leading to elevated intraluminal pressures in the pulmonary vasculature primarily of the small, muscular arteries (Chan and Vanhoutte, 2013). Chronic hypoxia entrenches structural and functional changes in the muscular pulmonary arteries that associate with smooth muscle cell (SMC) and endothelial cell (EC) proliferation and possible thrombosis. These changes are believed to be mediated by EC dysfunction or insult resulting in altered homeostasis between vasodilators and vasoconstrictors, growth inhibitors and mitogenic factors, and antithrombotic and prothrombotic factors (Farber and Loscalzo, 2004). That is, hypoxia alters the transcriptome of vascular cells altering extracellular matrix within evolving states defined by the relative changes in vasodilatation and vasoconstriction (Chan and Vanhoutte, 2013).

Arterial stiffening has gained attention as clinically relevant to changes in vascular biology that drive chronic conditions (Triposkiadis et al., 2019). It can be estimated by the so-called pulse wave velocity (PWV), that is, the speed of propagation of a wave of pressure and flow (pulse) of blood that travels along the pulmonary arteries with each beat of the right ventricle. As the wave travels distally (forward), a portion is reflected proximally (backward) due to interactions with the tapering and branching vessels through which the pulse travels. More elastic arteries distend and absorb the energy of the distally traveling wave, resulting in decreased PWV in the distal direction and less wave reflection in the proximal direction toward the right ventricle. Typically, reflected pulse waves in the pulmonary artery return to the right ventricle in late systole or early diastole. Stiffer arteries distend less and result in greater PWV distally and an increased reflection of the pulse wave proximally toward the right ventricle (Castelain et al., 2001; Lau et al., 2014). This results in greater magnitude pressure waves reaching smaller arteries and arterioles and thus distal tissues, which are not accustomed to high pulse pressures (Castelain et al., 2001). Increased PWV as produced by large arterial stiffening has been shown to induce pro-inflammatory and pro-proliferative responses in pulmonary arterial EC *in vitro* and in patients with pulmonary arterial hypertension (Li et al., 2009; Tan et al., 2014). Reflected waves also tend to arrive back to the right ventricle earlier and with greater magnitude due to pulmonary arterial stiffening. This results in an increased afterload against which the right ventricle must contract and to which the right ventricle will adapt and remodel (Castelain et al., 2001; Silva et al., 2017). Although it is axiomatic that many changes in the pulmonary vasculature that manifest at a clinical scale and determine particular therapeutic strategies result from underlying microstructural changes that in turn result from transcriptional changes that define changing vascular cell phenotypes, prior studies have focused primarily on changes at individual scales rather than integrative changes across scales. The goal of this paper, therefore, is to propose a different strategy, one that exploits recent advances in *in vivo* measurements in mice, *ex vivo* biomechanical phenotyping, microstructural assessments under *in vivo* relevant multiaxial loads, standard histology, and single cell RNA sequencing (scRNA-seq) to identify the transcriptional changes in individual cell types that drive adverse pulmonary vascular remodeling during periods of hypoxia. We submit that this multi-scale approach will not only advance the utility of diverse mouse models, but also facilitate translation of multiple methods and data analysis to investigation of the human pulmonary artery in health and diverse cardiopulmonary diseases, including pulmonary arterial hypertension, congenital heart defects, and COPD.

## Materials and Methods

**FIGURE 1:**
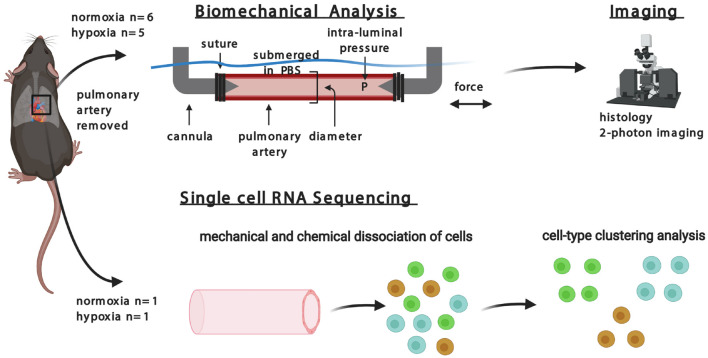
Multi-disciplinary approach to investigating hypoxia in pulmonary artery of mice.

The overall design of this study is summarized in Figure 1. Following surgical removal of the right pulmonary artery from mice, vessels were subjected either to *ex vivo* biomechanical measurements that maintained cell viability, followed by multiphoton microscopy and standard histology, or to isolation of cells for scRNA-seq to identify transcriptional changes. Data were collected and quantified separately for mice exposed to normoxic or hypoxic conditions. The study was approved by Yale University Institutional Animal Care and Use Committee.

### Hypoxic Exposure

Thirteen adult male mice (C57BL/6J; 14–20 weeks old, Jackson Laboratory, Bar Harbor, ME, United States) were housed in an antigen-free and virus-free animal care facility under a 12-h light and dark cycle. Six mice were exposed to hypoxic conditions by placing them in a plexiglass chamber connected to OxyCycler Models A420OC and AT42CO gas controllers and analyzers (BioSpherix, NY, United States). This gas control delivery system regulated the flow of room air, N_2_, and O_2_ into the chamber to create and maintain prescribed fraction inspired O2 (FiO_2_) levels. Persistent hypoxia defined by a FiO_2_ of 10% was maintained for 3–6 weeks at 24 h/day. Seven age matched C57BL6J mice were kept in similar conditions but exposed to room air (∼FiO_2_ 20%) as controls. Both groups of mice were fed a standard rodent chow and had free access to water. Animals were euthanized with overdose of urethane by intraperitoneal injection followed by exsanguination, followed by harvest of the hearts, lungs, and pulmonary arteries.

### Biomechanical Testing

Specimens were excised from the main pulmonary artery to the first branch of the right pulmonary artery (RPA) and prepared as previously described (Ramachandra and Humphrey, 2019). After flushing of blood with a Hanks buffered physiologic solution (Hanks and Wallace, 1949), perivascular tissue and fat were gently removed, and the left pulmonary artery and small branch vessels were ligated with suture. The RPA was cannulated on custom glass micropipettes and secured beyond the main pulmonary artery with ligature on one end and the first branch of the pulmonary at the other end. The specimen was submerged in a 37°C bath of Krebs-Ringer solution perfused with 95% O_2_/5% CO_2_. The specimens were tested using a custom computer-controlled testing device (Gleason and Humphrey, 2004), with an active protocol followed by a passive protocol (*n* = 5 for hypoxia, *n* = 6 for normoxia). Active testing focused primarily on SMC contractile responses near *in vivo* conditions, that is, a mean distending pressure of 15 mmHg and the specimen-specific value of *in vivo* axial stretch as previously described (Murtada et al., 2016; Caulk et al., 2019; Ramachandra and Humphrey, 2019). Such protocols are much more relevant physiologically than the standard “ring-tests” performed with uniaxial myographs. First, however, the SMCs were conditioned at 10 mmHg and an axial stretch of 1.1 (relative to the unloaded length) by contracting the vessel with 100 mM KCl. Axial stretch was subsequently increased in steps of 0.01 and pressure in steps of 1 mmHg to avoid sudden large deformations, which could compromise the viability of the SMCs and ECs. Following conditioning, the vessels were contracted with 100 mM KCl and then 1 mM phenylephrine (PE), both at 15 mmHg and the *in vivo* axial stretch. This protocol consisted of 5 min of equilibration, 15 min of contraction, and 10 min relaxation following washout of the vaso-stimulant with a fresh Krebs-Ringer solution. EC testing immediately followed maximal SMC contraction with PE without vaso-stimulant washout. Specimens were exposed to 10 μM acetylcholine (ACh) followed by inhibition of eNOS release by 1 mM N(gamma)-nitro-L-arginine methyl ester (L-NAME). Arterial diameter is regulated by the combined activities of EC and SMC; therefore, inhibition of EC secretion of vasodilatory molecules such as nitric oxide enables determination of maximal SMC contractility (Murtada and Humphrey, 2018).

### Multiphoton Microscopy

A Titanium-Sapphire Laser (Chameleon Vision II, Coherent) was used to image representative regions of pulmonary arteries at *in vivo* relevant loading conditions (*in vivo* stretches and pressures identical to those used during passive mechanical testing, described below). A LaVision Biotec TriMScope microscope was tuned at 840 nm and equipped with a water immersion 20× objective lens (NA. 0.95). The backward scattering second harmonic generation signal from fibrillar collagens was detected within the wavelength range 390–425 nm; the autofluorescent signal arising from elastin was detected at 500–550 nm, and the fluorescent signal of cell nuclei labeled with Syto red stain was detected above 550 nm. An in-plane field of view (axial-circumferential plane) of 500 μm × 500 μm and a volume of about 0.05 mm^3^ were used; this provides a much greater volume of tissue for imaging than via standard histology and hence averaging over significantly greater numbers of cells and extracellular matrix. The in-plane resolution was 0.48 μm/pixel and the out-of-plane (radial direction) step size was 1 μm/pixel. 3D images acquired concurrently for the three signals (collagen, elastin, and cell nuclei) were post-processed using MATLAB R2019b and ImageJ 1.53a. The first processing step relied on the near cylindrical shape of the samples to fit a circle to the two-dimensional mid-thickness profile of the arterial wall and transform each circumferential-radial slice of the 3D images from Cartesian to polar coordinates (angle and radius). This allowed a layer-specific microstructural analysis to focus on collagen fiber alignment and cell volume density analyses, as described in previously (Cavinato et al., 2020, 2021).

### Histology

Following biaxial testing and multiphoton imaging, the specimens were fixed in 10% neutral buffered formalin and stored in 70% ethanol at 4°C for histology. After embedding in paraffin, they were sectioned (5 μm thickness) and radial-circumferential cross-sections (planes) were stained with Verhoeff Van Giesen (VVG), Masson’s Trichrome (MTC) or Movat pentachrome. Details of image quantification can be found elsewhere (Bersi et al., 2017). Briefly, each section was imaged with an Olympus BX/51 microscope using an Olympus DP70 digital camera under a 20× magnification objective. Complete cross-sections were obtained by stitching together sub-images with Image Composite Editor software (Microsoft Research). The stitched images were subsequently analyzed using custom MATLAB scripts. Briefly, following background subtraction and pixel-based thresholding, area fractions for elastin (from VVG) and cytoplasm (from MTC) were computed as the ratio of pixels corresponding to a stain divided by the total number of pixels in the image. Because MTC can overstain collagen, its area fraction was computed as 1 - area fraction of elastin plus cytoplasm, with GAG content assumed negligible (Ferruzzi et al., 2015, 2018) as confirmed with Movat Pentachrome staining (data not shown). Three sections were analyzed per vessel per stain.

### Material Characterization

We used a 2-D formulation to model the passive mechanical behavior since residual stresses tend to homogenize the stress field, thereby rendering mean values as good estimates of overall wall stress (Humphrey, 2013). Mean stresses along the circumferential (θ) and axial (z) directions are,


$$\sigma_{\theta }=\frac{Pr_{i}}{r_{o}-r_{i}},\text{and}\sigma_{z}=\frac{f_{T}+P\pi r_{i}^{2}}{\pi \left(r_{o}^{2}-r_{i}^{2}\right)},$$

where *P* is the transmural pressure, *r_i_* internal radius, *r_o_* outer radius, and *f_T_* the transducer measured axial force. Under the assumption of incompressibility, inner radius $r_{i}=\sqrt{r_{o}^{2}-\bar{V}/\pi l}$, where *l* is the instantaneous length between the ligatures securing the vessel to the micropipettes and $\bar{V}$ is the volume of the vessel in the unloaded state; $\bar{V}=\pi L\left(OD^{2}-ID^{2}\right)/4$ where *L* is the unloaded length, *OD* the unloaded outer diameter, and *ID = OD −2H*, the unloaded inner diameter.

A pseudoelastic constitutive formulation, which has successfully described passive biaxial behaviors of pulmonary arteries (Ramachandra and Humphrey, 2019), modeled the passive mechanical behavior in terms of a stored energy function, *W*. Associated wall stress and material stiffness can be computed from first and second derivatives of *W* with respect to an appropriate deformation metric. Based on success of prior work, we let


$$W=\frac{c}{2}\left(I_{c}-3\right)+\sum_{i=1}^{4}\frac{c_{1}^{i}}{4c_{2}^{i}}\left\{exp\left[c_{2}^{i}{\left(IV_{c}^{i}-1\right)}^{2}\right]-1\right\},$$

where *c*, $c_{1}^{i}$ and $c_{2}^{i}$ are material parameters; *i* = 1, 2, 3, 4 represent four collagen-dominated families of fibers along axial, circumferential, and two symmetric diagonal directions, respectively. *I_c_* is the first invariant of right Cauchy-Green tensor (=$\lambda_{\theta }^{2}+\lambda_{z}^{2}+1/\lambda_{\theta }^{2}\lambda_{z}^{2}$) and $IV_{c}^{i}$ is the square of the stretch of the *i*^*th*^ fiber family (=$\lambda_{\theta }^{2}sin^{2}\alpha_{0}^{i}+\lambda_{z}^{2}cos^{2}\alpha_{0}^{i}$); $\alpha_{0}^{i}$ is the fiber angle relative to axial direction in the reference configuration. λ_θ_ = (*r*_*i*_ + *r*_*o*_)/ 2ρ_*mid*_, λ_*z*_ = *l*/*L* are the mean circumferential and axial stretch, and ρ_*mid*_ is the unloaded mid-wall radius. The best-fit values of the material parameters and the fiber angle were determined via nonlinear regression of biaxial data from all seven passive protocols. More details on parameter estimation can be found elsewhere (Ramachandra and Humphrey, 2019).

Pulse wave velocity depends on both the geometry and mechanical properties of the arteries and can be well-approximated based on the material stiffness or distensibility of the arterial walls, namely


$$\text{PWV}=\sqrt{\frac{1}{\rho \cdot D}}\left(Bramwell-Hillequation\right),$$

where ρ is the mass density of the contained fluid (approximately 1,050 kg/m^3^) and *D* is the distensibility coefficient (in Pa^–1^or kg^–1^m⋅s^2^) determined by the normalized change in arterial diameter from end-diastole to end-systole divided by the change in end-diastolic and end-systolic pressures (Bramwell and Hill, 1922). PWV in the pulmonary artery and its effects on right ventricular hemodynamics can thus be measured non-invasively using the aforementioned variables (Peng et al., 2006; Sanz et al., 2008; Gupta et al., 2018).

### Statistics

For variables with only two categories, differences between hypoxic and normoxic groups in morphological, histological, mechanical, and contractile properties were determined by a two-tailed, unpaired *t* test. For categorical variables with more than two categories, levels of stretch or pressure were compared using a two-factor analysis of variance (ANOVA). A global test across all levels was performed and then pair-wise comparisons were conducted with post-hoc tests using Bonferroni adjustment. A *p <* 0.05 level of significance was used, with data reported as mean ± standard error from the mean (SEM).

### Single Cell RNA Sequencing

Viable cells from the main pulmonary arteries of one mouse exposed to hypoxic conditions for six weeks and one age-matched normoxic mouse were analyzed. Following euthanasia, the heart, lungs, and pulmonary arteries were excised with surrounding tissue. The pulmonary arteries were chopped mechanically and placed in 1 mg/ml collagenase (Roche) and 3 U/ml elastase (Worthington). Following cellular dissociation, we barcoded unique mRNA molecules of each cell using our 10× Genomics Chromium platform (3’ v3.1 kit), a droplet-based microfluidic system, and performed reverse transcription, cDNA amplification, fragmentation, adaptor ligation, and sample index PCR according to the manufacturer’s protocol. High sensitivity DNA bioanalyzer traces of cDNA after barcoding and of the final cDNA library were evaluated for quality control. The final cDNA libraries were sequenced on a HiSeq 4000 Illumina platform in our core facility aiming for 150 million reads per library. Raw sequencing reads were demultiplexed based on sample index adaptors, which were added during the last step of cDNA library preparation. Possible adaptor and/or primer contamination were removed using Cutadapt. We processed the RNA-sequence data using the scRNA-seq implementation of STAR (STARsolo), where reads were mapped to the murine reference genome GRCm38 release M22 (GRCm38.p6), collapsed and counted, and summarized to a gene expression matrix. Data were analyzed and visualized using the R packages Seurat (Stuart et al., 2019). Specifically, we clustered the cellular transcriptomes and visualized them in a uniform manifold approximation and projection (UMAP) space to delineate cell types. To identify aberrant gene expression profiles in cellular subpopulations, we established differentially expressed genes between normoxic and hypoxic cells using the non-parametric Wilcoxon rank sum test with Bonferroni adjustment of multiple testing. We used connectomic analyses (R package “Connectome”) (Raredon et al., 2019) to identify conserved patterns of cell-cell cross-talk between the ECs, SMCs and fibroblasts in the pulmonary artery (Raredon et al., 2019; Browaeys et al., 2020). Connectomtic analysis uses an algorithm to visualize intercellular signaling using ligand-receptor mapping. It filters single-cell data to identify the distribution of ligand and receptor z-scores and percent expression to identify cell-type specific communication patterns (edges) with increased statistical confidence. The thickness of the edges corresponds to the z-score (Raredon et al., 2019). Enriched terms analyses of phenotype (using MGI Mammalian Phenotype Level 4 2019 database) and signaling pathways (using WikiPathways 2019 Mouse database) were based on differentially expressed genes when comparing hypoxic with normoxic genetic expression (Chen et al., 2013; Kuleshov et al., 2016). The top results of the gene-set libraries of the collective functions of gene lists generated from the gene list of our single cell sequencing experiments were displayed and plotted as bar charts to show the top enriched terms in the chosen library based on their *p*-value (plotted as −log_10_*p*-value).

## Results

### Hypoxia Alters Tissue-Level Properties

**FIGURE 2:**
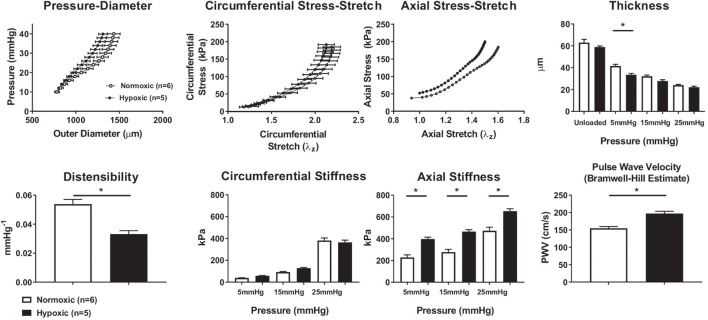
Mechanical properties of normoxic and hypoxic mouse pulmonary arteries. **(Top row)** Data collected (pressure, arterial diameter, stretch) or calculated (stress) from cannulated normoxic (*n* = 6) and hypoxic (*n* = 5) right pulmonary arteries of C57BL6J mice using a computer-controlled testing device. Pressure-diameter measurements were at vessel-specific optimized axial stretch (mean ± SEM). Stress-stretch measurements were taken with the vessel at 15 mmHg. Wall thickness measurements for normoxic (*n* = 6) and hypoxic (*n* = 5) right pulmonary artery vessels at various pressures. Unloaded (zero mmHg intraluminal pressure) wall thickness was measured by histologic analysis and MatLab. Loaded (5, 15, 25 mmHg) wall thickness measurements were calculated based on mechanical measurements. Despite some differences at individual pressures, there is no overall difference in thickness of normoxic and hypoxic vessels. **(Bottom row)** Mechanical properties calculated from mechanical testing and fit to the four-fiber constitutive relation, including distensibility, circumferential stiffness, axial stiffness, and PWV. There is a significant decrease in distensibility observed in hypoxic vessels. The circumferential stiffness of hypoxic and normoxic pulmonary arteries is similar at lower pressures; however, at 25 mmHg, the circumferential wall stress in hypoxic pulmonary arteries tends to decrease. The axial stiffness calculated for hypoxic vessels is greater than normoxic vessels at diastolic, mean, or systolic pressures (5, 15, 25 mmHg, respectively). The changes in structural and mechanical properties of the arterial wall and distensibility due to hypoxic exposure result in a significant increase (*p* < 0.05) in PWV of the right pulmonary artery of mice at 15 mmHg. *p < 0.05.

Biomechanical phenotyping of normoxic (*n* = 6) and hypoxic (*n* = 5) RPAs revealed a general structural stiffening in hypoxia despite modest changes in wall thickness (Figure 2). The mean pressure-diameter findings are shown at the vessel-specific optimal, or *in vivo*, axial stretch and similarly for the circumferential stress-stretch findings; the axial stress-stretch findings are shown at a common pressure of 15 mmHg. The increased structural stiffness was reflected further by a significant decrease in distensibility and associated increase in the calculated PWV. The modest decrease in wall thickness in hypoxia (inferred during *ex vivo* testing and confirmed via multiphoton microscopy; Supplementary Figure 1) implicated an increase in material stiffness in hypoxia, which in turn suggested a change in extracellular matrix. Material stiffness and elastically stored energy were computed using the four-fiber family constitutive relation, with associated best-fit material parameters (Supplementary Table 1). Calculated values of biaxial material stiffness (circumferential and axial) were greater for hypoxic than for normoxic vessels in the axial direction alone at diastolic, mean, and systolic pressures (5, 15, 25 mmHg, respectively), as shown in Figure 2. Hypoxia did not change the elastically stored energy at diastolic or mean pressures but did decrease it at systolic pressures (Supplementary Figure 2).

### Hypoxia Induces Microstructural Changes, Notably Collagen Remodeling

**FIGURE 3:**
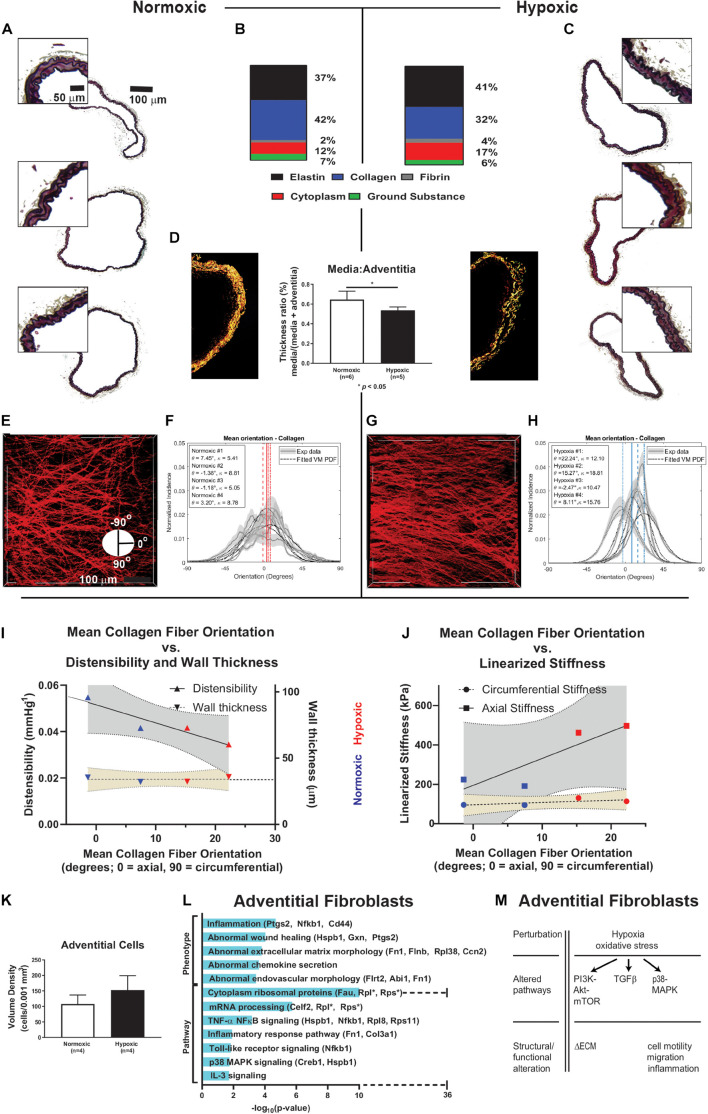
Composition analyses, collagen orientation correlation with stiffness. **(A,C)** Paraffin embedded, Movat-stained cross-sectional imaging of representative pulmonary arteries from normoxic mice (*n* = 3) and hypoxic mice (*n* = 3). The complete pulmonary arteries are composite images of 20× magnification stitched together using Microsoft Image Editor. Small portions of the wall for these samples were enlarged with equal proportion for closer visual examination and comparison. **(B)** The area fractions for elastin (from VVG), cytoplasm (from MTC), and ground substance and fibrin (from Movat) were computed based on *n* = 5 samples for each stain. Area fraction of collagen was computed as 1 - area fraction of elastin plus cytoplasm plus ground substance. **(D)** The media to adventitia thickness ratio was calculated using picosirius red staining and imaged using polarized light to identify media and adventitial collagen components, revealing significant decrease in medial collagen in hypoxic samples (*n* = 5) compared to normoxic samples (*n* = 6), *p* < 0.05. **(E–H)** Representative 2-photon images of collagen fibers (red) at 15 mmHg for a normoxic and hypoxic vessel. Collagen fiber alignment (κ) and orientation (θ) analyses for normoxic (*n* = 4) and hypoxic (*n* = 4) vessels at 15 mmHg. Collagen fiber alignment is represented by the width of the collagen fiber orientation distribution, κ, where κ and width of distribution are inversely related. Collagen fiber orientation shows distribution of collagen fiber alignment ranging from the axial direction (0 degrees, running the length of the pulmonary artery) to the circumferential direction (-90 or 90 degrees, running around the circumference of the pulmonary artery and orthogonal the axial direction). Hypoxic exposure causes a circumferential shift in collagen fibers. **(I,J)** Linear least squares fit of data with 95% confidence bands for distensibility (*R*^2^ = 0.88), arterial wall thickness (*R*^2^ = 0.07), and linearized axial stiffness (*R*^2^ = 0.77) and circumferential stiffness (*R*^2^ = 0.48) with respect to mean collagen fiber orientation. Material stiffness of pulmonary arteries correlates with collagen fiber alignment, arterial wall thickness does not. Circumferential orientation of collagen fibers directly correlates with axial stiffness. **(K)** 2-photon imaging revealed an increase in the cell density of adventitial cells of hypoxic pulmonary arteries (*n* = 4) compared to normoxic (*n* = 4). Cell numbers were then normalized by a volume of 0.001 mm^3^ to allow consistent comparisons. We found similar results in H&E-stained paraffin samples (normoxic *n* = 6, and hypoxic *n* = 5) as shown in Supplementary Figure 5. **(L)** Enriched terms analysis of phenotype (using MGI Mammalian Phenotype Level 4 2019 database) and signaling pathways (using WikiPathways 2019 Mouse database). The bar chart shows the top enriched terms in the chosen library based on their *p*-value (plotted as −log_10_*p*-value). Fibroblast differential gene expression due to hypoxic exposure is associated with phenotypic changes in extracellular matrix previously associated with inflammation and endovascular morphology. Pathways associated with the differentially expressed genes observed due to hypoxia include TNF-kB, TLR signaling, p38-MAPK, and IL-3. There is a large increase in the number of ribosomal genes expressed in response to hypoxic exposure. **(M)** Schematic summary of hypoxia-induced alterations of cellular pathways and function in fibroblasts based on transcriptomic and functional data. ECM = extracellular matrix.

Wall composition and characteristics of intramural collagen are shown in Figure 3 for representative RPAs from normoxic and hypoxic mice. Movat-stained cross-sections of normoxic and hypoxic vessels suggested no major changes in composition (Figures 3A–C). Area fractions for normoxic and hypoxic RPAs were computed for elastin (37 ± 3.5% vs. 41 ± 4.4%, respectively, *p* = 0.43), collagen (42 ± 8.0% vs. 32 ± 7.8%, *p* = 0.40), cytoplasm (12% fraction ± 2.9% vs. 17 ± 3.3%, *p* = 0.27), ground substance (7% fraction ± 0.9% vs. 6 ± 0.8%, *p* = 0.20), and fibrin (2% fraction ± 0.3 vs. 4 ± 1%, *p* = 0.14). Pico-sirius red stained sections imaged with polarized light (Figure 3D) revealed, however, that medial collagen was reduced in hypoxic compared to normoxic vessels (*p <* 0.05). Alignment and orientation of collagen was assessed using multiphoton imaging, with characteristic images shown at 15 mmHg for both groups (Figures 3E–H); additional images are included in Supplementary Figure 3 and Supplementary Material. The mean orientation of collagen fibers (primarily adventitial) was less axially directed for the hypoxic (10.8° ± 5.3°) than the normoxic (2.0° ± 2.1°) vessels (*p* = 0.20). Collagen fiber alignment was significantly greater for the hypoxic (κ = 14.3 ± 1.9) than the normoxic (κ = 7.0 ± 1.0) vessels (*p* = 0.02), where the parameter κ and width of distribution are inversely related (i.e., lower κ equals greater width of distribution and *vice versa*). To the author’s knowledge, these are the first published measurements of collagen fiber alignment in pulmonary arteries of mice. Note, too, that immunohistochemistry delineated changes in these fibrillar collagens by type, I versus III, revealing a decrease in the former and increase in the latter with hypoxia (Supplementary Figure 4).

Pearson correlation analysis revealed that collagen fiber orientations correlated inversely with distensibility (*R*^2^ = 0.88), but not wall thickness (*R*^2^ = 0.07), as shown in Figure 3I. Collagen fiber orientation also correlated directly with axial material stiffness (*R*^2^ = 0.77) but less so with circumferential stiffness (*R*^2^ = 0.48), as shown in Figure 3J. Multiphoton imaging also revealed an increase in the density of adventitial cells in the hypoxic compared with the normoxic arteries (Figure 3K). Similar results were found in H&E-stained paraffin samples (Supplementary Figure 5), noting that the ratio of the medial : adventitial cross-sectional areas did not change (Supplementary Figure 6). Immunohistochemistry and immunofluorescence were used to confirm that the layers and morphology of cells of the intimal, medial, and adventitial layers were not co-located (Supplementary Figure 5). Recall that paraffin-embedded histological cross-sections are sampled at ∼5 μm increments along the length of the artery, whereas multiphoton imaging is along an entire 500 μm length. Noting that most of the collagen fibers were in the adventitia, enriched terms analysis of phenotype and signaling pathways are shown for fibroblasts in Figure 3L. The bar chart shows the top enriched term search results based on their *p*-value (plotted as −log_10_*p*-value). Differential gene expression in fibroblasts due to hypoxia associated with phenotypic changes in extracellular matrix previously associated with inflammation and endovascular morphology. Pathways associated with the differentially expressed genes observed due to hypoxia included PI3K-Akt-mTOR, TGFβ, and p38-MAPK, as highlighted in Figure 3M. Differentially expressed genes associated with NF-kB, TLR signaling, and IL-3 signaling were also observed. There was a large increase in the number of ribosomal genes expressed in response to hypoxic exposure.

### Hypoxia Alters Vasoactive Function

**FIGURE 4:**
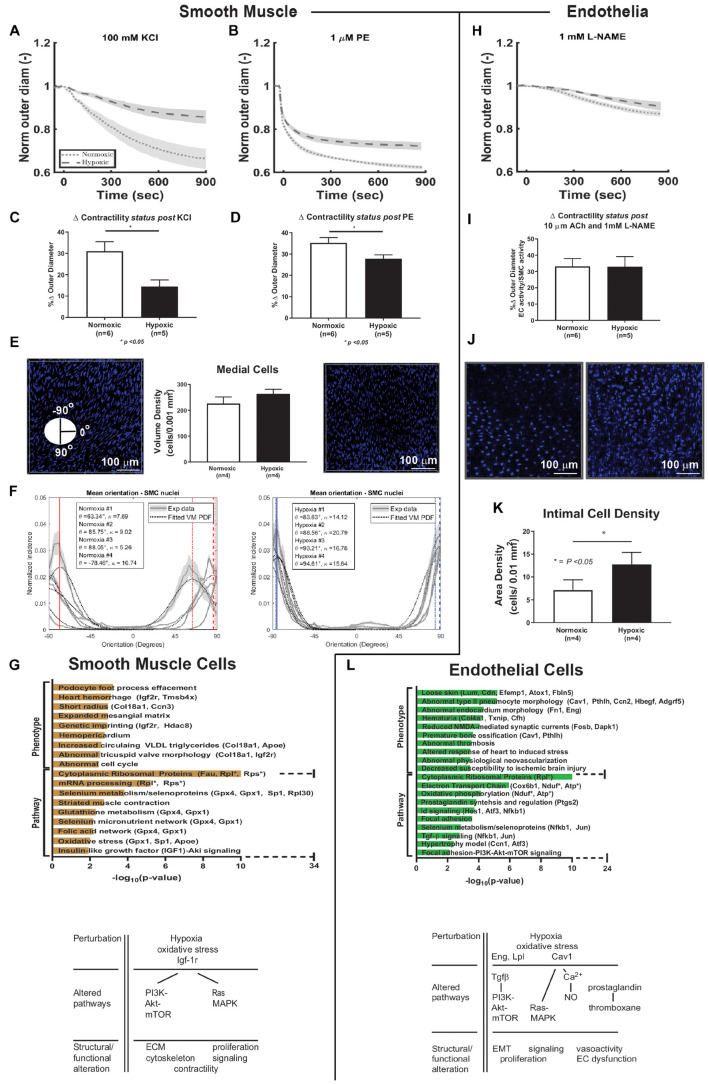
Smooth muscle cell and endothelial cell functional-transcriptomics. **(A,B)** Vaso-constriction response to vasoactive substances, potassium chloride (KCl) and phenylephrine (PE), as a function of time plotted as mean (dashed and dotted lines) ± standard error from the mean (shade) for normoxic (dotted, *n* = 6) and hypoxic (dashed, *n* = 5) samples. Vessels were maintained at a fixed pressure (15 mmHg) and specimen-specific optimized axial stretch. **(C,D)** Percent change in contractility based on normalized outer diameter change at the end of 15 min exposure to KCl and PE. There is a significant decrease in contractility of hypoxic vessels compared to normoxic vessels for both vasoactive substances (*p* < 0.05). **(E)** Cell quantification in medial layers. Cellular density measurements were made using 2-photon imaging of normoxic (*n* = 4) and hypoxic (*n* = 4) vessels and normalized by a volume of 0.001 mm^3^. Representative images from 2-photon imaging are shown. Similar measurements were made on the number of cells per layer based on counting nuclei using H&E stained paraffin-embedded cross-sections of right pulmonary arteries using light microscopy at 20× magnification as shown in Supplementary Figure 5. An advantage of 2-photon imaging is its ability to samples larger regions of the vessel than histology, which only samples one cross-section of the artery at a time. **(F)** Analysis of SMC orientation based on 2-photon imaging reveals increased alignment of these cells following hypoxic exposure and a shift in orientation toward the circumferential direction similar to the phenomenon that we observed in collagen fibers in hypoxic pulmonary arteries. **(G)** Enriched terms analysis of phenotype (using MGI Mammalian Phenotype Level 4 2019 database) and signaling pathways (using WikiPathways 2019 Mouse database). The bar chart shows the top enriched terms in the chosen library based on their *p*-value (plotted as −log_10_*p*-value). SMC differential gene expression due to hypoxic exposure is associated with phenotypic changes in morphology, matrix, and cell cycle. Pathways associated with the differentially expressed genes observed due to hypoxia include selenoproteins, proteins of myocontractility, and oxidative stress. There is a large increase in the number of ribosomal genes expressed in response to hypoxic exposure. Hypoxia-induced alterations of cellular pathways and function in SMC are summarized in the schematic diagram. **(H)** Vaso-dilatation measured following exposure to acetylcholine (Ach) followed by inhibition of eNOS release by L-NAME. Effects of L-NAME on vessel outer diameter over time plotted as mean (dashed and dotted lines) ± standard error from the mean (shade) for normoxic (dotted, *n* = 6) and hypoxic (dashed, *n* = 5) samples. Vessels were maintained at a fixed pressure (15 mmHg) and specimen-specific optimized axial stretch. **(I)** Percent change in vasoactivity based on normalized outer diameter change at the end of 15 min exposure to L-NAME. There was no significant change when comparing hypoxic and normoxic vessels. **(J)** Representative images from 2-photon imaging of the intimal layer. **(K)** Cell quantification of intimal layer. Cellular density was computed as number of cells per surface area and normalized by an area of 0.01 mm^2^ from 2-photon images. **(L)** Enriched terms analysis of phenotype (using MGI Mammalian Phenotype Level 4 2019 database) and signaling pathways (using WikiPathways 2019 Mouse database). The bar chart shows the top enriched terms in the chosen library based on their *p*-value (plotted as −log_10_*p*-value). EC differential gene expression due to hypoxic exposure is associated with phenotypic changes in focal adhesion, endovascular morphology, thrombosis, neovascularization, and protection against ischemia. Pathways associated with the differentially expressed genes observed due to hypoxia include oxidative phosphorylation, prostaglandins, NFkB, TGFb, and PI3K-Akt-mTOR signaling. There is a large increase in the number of ribosomal genes expressed in response to hypoxic exposure. Hypoxia-induced alterations of cellular pathways and function in EC are summarized in the schematic diagram. ECM = extracelluluar matrix. EMT = endothelial-mesenchymal transitioning. *p < 0.05.

SMCs endow the pulmonary artery with the ability to vasoconstrict and vasodilate, which were assessed as responses to vasoactive substances at a fixed pressure (15 mmHg) and optimized specimen-specific axial stretch (Figure 4), both over time (panels A and B) and as a percent change based on normalized outer diameter at the end of 15 minutes (C and D). The normalized reduction in outer diameter during vasoconstriction (KCl and PE) of control mice (normoxia) was 31 and 35%, respectively, at 15 min, similar to previous findings (Ramachandra and Humphrey, 2019), while hypoxic exposure (10% FiO_2_ for 3–6 weeks) resulted in a statistically significant decrease (*p* < 0.05) in this measure of contractility *ex vivo* for both KCl (14%) and PE (28%). Multiphoton imaging of the SMCs yet revealed that their mean orientation was more circumferential (θ–90°) in hypoxic (84°–88°) than in normoxic (63°–86°) vessels (panel E). SMC alignment was also greater in hypoxic (κ = 14.12–20.79) than in normoxic (κ = 7.59–9.03) vessels (panel F). Enriched terms analysis of phenotype and signaling pathways are shown for SMC cells (panel G). SMC differential gene expression due to hypoxic exposure associated with phenotypic changes in morphology, matrix, and cell cycle. Pathways associated with the differentially expressed genes observed due to hypoxia include selenoproteins, proteins of myocontractility, and oxidative stress. Differentially expressed genes associated with PI3K-Akt-mTOR and Ras-MAPK intracellular pathways, as highlighted in Figure 4G. There was a large increase in the number of ribosomal genes expressed in response to hypoxic exposure.

Endothelial cell-dependent vaso-dilatation was measured in response to acetylcholine (Ach), with further functional assessment following eNOS inhibition with L-NAME, the latter shown in panel H. The normalized baseline outer diameter in this case (1.0) is the vessel-specific outer diameter following maximal contraction with PE and after 10 minutes exposure to 10 μM Ach. Panel I reveals no significant hypoxia-induced change in EC induced vasodilatation *ex vivo*. The authors are not aware of previously published data regarding EC function in the *ex vivo* mouse pulmonary artery. Multiphoton imaging further revealed an increase in the density of intimal cells of hypoxic compared with normoxic vessels (Panel J, images, and panel K, quantitative). Results from H&E-stained paraffin-embedded sections were consistent (Supplementary Figure 5). Enriched terms analysis of phenotype and signaling pathways for ECs (panel L) revealed that differential gene expression due to hypoxic exposure associated with phenotypic changes in focal adhesion, endovascular morphology, thrombosis, neovascularization, and protection against ischemia. Pathways associated with the differentially expressed genes observed due to hypoxia include TGFβ and PI3K-Akt-mTOR signaling and endothelial processes such as thrombosis (prostaglandins and thromboxane), oxidative phosphorylation, and vasoactivity. These changes and associated structural or functional alterations are summarized in Figure 4L. There was a large increase in the number of ribosomal genes expressed in response to hypoxic exposure.

### Hypoxia Resulted in Hundreds of Differentially Expressed Genes in Resident Cell Types

**FIGURE 5:**
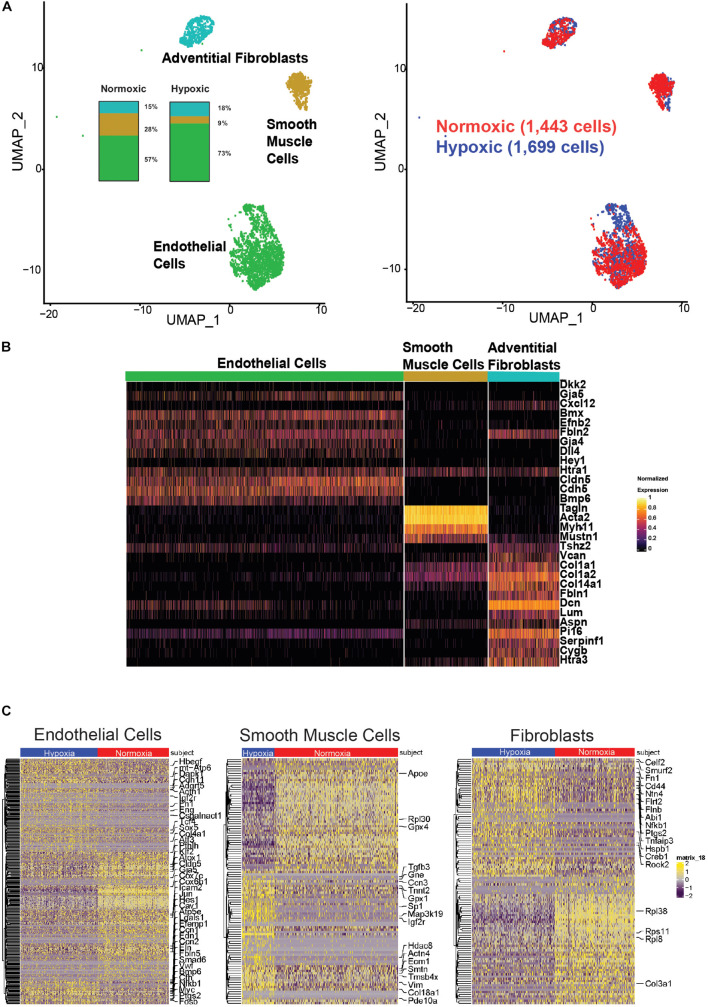
Single cell RNA sequencing of mouse pulmonary artery resident cells. **(A)** Uniform Manifold Approximation and Projection (UMAP) representation of cells clustered by expressed genes. About 1,443 cells from hypoxic PA [1,053 endothelial cells (EC), 127 smooth muscle cells (SMC), 263 adventitial fibroblasts (FB)]. About 1,699 cells from normoxic PA (967 EC, 479 SMC, 253 FB). Although there are similar number of cells overall between normoxia and hypoxia samples, there is a decrease in the proportion of SMC in the hypoxia sample and an increase in the proportion of EC. **(B)** Heatmap identifies cell markers used to identify PA resident cell types. **(C)** Heat maps of differentially expressed genes by cell-type. EC: 331 differentially expressed genes in were found in hypoxic EC. Hypoxic arterial EC show higher expression of genes associated with hypoxia-induced proteins, cellular differentiation, proliferation and angiogenesis and lower expression of genes associated with oxidative respiration metabolism. Hypoxic EC are associated with lower expressions of intercellular junction proteins with higher expression of cytoskeletal and extracellular genes. There is higher expression of genes associated with endothelial-mesenchymal transitioning (Medici and Kalluri, 2012; Chen et al., 2016) and a higher expression of genes associated with chemotaxis and vasodilators. SMC: 107 differentially expressed genes were found in hypoxic SMC. Hypoxic SMCs show a higher expression of genes associated with cytoskeletal rearrangement (van der Loop et al., 1996; Worth et al., 2001; Chen et al., 2016; Pedroza Albert et al., 2020) and cell signaling. Fibroblasts: 113 differentially expressed genes were found in hypoxic fibroblasts. Fibroblasts show a higher expression of genes associated with cellular adhesion and migration, degradation of extracellular matrix and the ubiquitination-proteosome system.

We identified 3,142 resident cells from a segment of the pulmonary artery from one mouse exposed to hypoxic conditions (FiO_2_ 10%) for six weeks and one age-matched control mouse exposed to normoxic conditions (FiO_2_ 21%). These cells were represented on a uniformed manifold approximation and projection (UMAP) of cells clustered by genetic markers and cellular origin for normoxia versus hypoxia (Figure 5). Identification of resident cell types was based on their expression of marker genes as shown in Figure 5B: endothelial cells (*Dkk2, Gja5, Cxcl12, Bmx, Efnb2, Fbln2, Gja4, Dll4, Hey1, Htra1, Cldn5, Cdh5*, and *Bmp6*), smooth muscle cells (*Tagln, Acta2, Myh11, Mustn1*), and fibroblasts (*Tshz2, Vcan, Col1a1, Col1a2, Col14a1, Fbln1, Dcn, Lum, Aspn, Pi16, Serpinf1, Cygb*, and *Htra3*). There was a decreased proportion of SMCs and increased proportion of ECs in the hypoxic sample, as shown in the inset of Figure 5A. Figure 5C shows heat maps for differentially expressed genes in ECs, SMCs, and fibroblasts. There were 331 differentially expressed genes in hypoxic ECs, with higher expression associated with hypoxia-induced proteins for cellular differentiation and proliferation and angiogenesis such as *Igfb7, Ifg1r, Igf2r, Myc, Sox5, Lgals1* and lower expression of genes associated with oxidative respiration metabolism such as *Atp5mpl, mt-Atp6Atp5e*, and *Cox7c*. Hypoxic ECs also had lower expressions of intercellular junction proteins such as *Icam2, Gja5, Cdh11*, and *Cldn5* with higher expression of cytoskeletal and extracellular proteins such as *Fbln5, Actn1, Eln, and Ccn2*, and *Csgalnact1*. There is higher expression of genes associated with endothelial-mesenchymal transitioning, such as *Klf2, Fn1, Bmp6, Smad6, Tcf4*, and *Eng* (Medici and Kalluri, 2012; Chen et al., 2016). Finally ECs had a higher expression of chemotactic proteins such *Vwf* and *Hbegf* in hypoxia as well as *Edn1*, which encodes endothelin-1, a potent vaso-constrictor. There were 107 differentially expressed genes in hypoxic SMCs, including a higher expression of genes associated with cytoskeletal rearrangement such as *Tgfb3, Vim, Actn4, Tnnt2, Col18a1, Ecm1*, and *Smtn* (van der Loop et al., 1996; Worth et al., 2001; Chen et al., 2016; Pedroza Albert et al., 2020) and cell signaling such as *Gne, Pde10a*, and *Map3k19*. Finally, there were 113 differentially expressed genes in hypoxic fibroblasts, with higher expression of genes associated with cellular adhesion and migration such as *Cd44, Rock2* and *Fn1* and the degradation of extracellular matrix and the ubiquitination-proteosome system such as *Smurf2, Tnfaip3, and Ntn4*. This is the first single cell RNA sequencing of mouse pulmonary arteries of which the authors are aware, noting that the induced hypoxia resulted in an increase in right ventricular pressure and cavity volume (Supplementary Figure 7).

**FIGURE 6:**
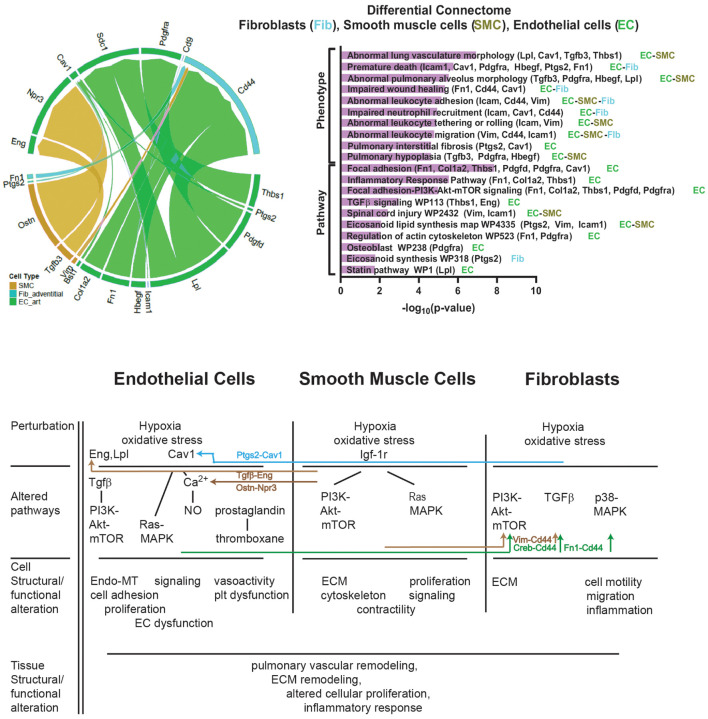
Hypoxia-normoxia differential connectome of mouse pulmonary artery resident cells. **(Left)** Differential connectome mapping comparing hypoxic and normoxic communication based on scRNAseq data to identify differences between hypoxic and normoxic cell-cell signaling. Differential connectomic analysis identifies and visualizes the major perturbed cell–cell communication pathways between single cell data from similar cell types under two different conditions, in this case hypoxia versus normoxia, using ligand-receptor connectomic analysis (Raredon et al., 2019). Connecomtic analysis is an algorithm to visualize intercellular signaling using ligand-receptor mapping. It filters single-cell data to identify the distribution of ligand and receptor z-scores and percentage expression to identify cell-type specific communication patterns (edges) with increased statistical confidence. The thickness of the edges is proportional to the ligand and receptor z-scores. The bottom half of the circle identifies the ligand gene and “sender” cell-type, the top half of the circle identifies the receptor gene and “receiver” cell-type. **(Right)** Enriched terms analysis of phenotype (using MGI Mammalian Phenotype Level 4 2019 database) and signaling pathways (using WikiPathways 2019 Mouse database) based on genes identified through differential connectome analysis. The bar chart shows the top enriched terms in the chosen library based on their *p*-value (plotted as −log_10_*p*-value). Genes associated with the identified phenotype or pathway are listed along with the cell-type in which these genes were found to be expressed. The differential connectome cell-cell signaling genes identified accurately represent observed phenomena and pathways associated with phenomena observed in our experiments including abnormal lung vasculature, fibrosis, alterations of cell cycle, regulation of actin cytoskeleton, TGF-beta signaling, and alterations in focal adhesion. Whether this type of analysis hold predictive abilities requires further investigation. **(Bottom)** Potential cell–cell interactions between based on the differential connectome demonstrating how differentially expressed genes from one cell type may influence gene expression and cellular pathways in another cell type. ECM = ECM = extracellular matrix. Endo-MT = endothelial-mesenchymal transitioning. EC = endothelial cell.

Differential connectomic analysis identifies changes in cell-type-specific molecular communications that occur among ECs, SMCs, and fibroblasts when comparing hypoxic and normoxic conditions based on previously described protein interactions (Figure 6). These results demonstrate how differentially expressed genes within one cell type may affect gene expression in another cell type, ultimately altering structure and function at the tissue level. *Fn1/Vim-Cd44* connections between ECs and fibroblasts in the RPA involves a pathway that has been associated with ECM-cell receptor interactions (Geng et al., 2019) and endothelial-to-mesenchymal transition (Endo-MT) (Goveia et al., 2020). The *Tgfb3-Eng* connection between SMCs and ECs is associated with EC adhesion and regulation of ECM formation (Cheifetz et al., 1992; Kaartinen et al., 1995). *Hbegf-Cd9* signaling seen in the connections between ECs and fibroblasts has previously been associated with preservation of membrane barrier function (Schenk et al., 2013) and enhancing cell viability (Takemura et al., 1999). Enriched terms analysis of phenotype and signaling pathways based on genes identified through differential connectome analysis revealed associations with abnormal lung vasculature, altered collagen deposition, alterations of cell cycle, regulation of actin cytoskeleton, TGF-beta signaling, and alterations in focal adhesion. This captures phenomena and pathways of importance that we observed in this study. These are the first time that these communications between the resident cell types of the mouse pulmonary arteries resulting from hypoxic exposure have been described in this manner.

## Discussion

This study reports previously undescribed changes in the orientation of collagen fibers and similarly SMCs in the mouse RPA following 3-6 weeks of hypoxic exposure, with associated structural stiffening and decreased contractility. Cell-specific differentially expressed genes suggested potential molecular mechanisms and pathways driving these changes.

That the pulmonary artery stiffening in hypoxia observed herein appears to result more from collagen remodeling than increased accumulation is in contrast to hypertension-induced stiffening of the aorta, the largest elastic artery of the systemic circulation, which results from increased collagen largely in the adventitia (Bersi et al., 2016, 2017; Oh Young et al., 2017). This finding has multiple implications, including differential species-dependent responses. Previous reports detail thickening of the branched pulmonary arteries from increased adventitial collagen in rats due to acute hypoxic exposure ranging from hours to 30 days (Fung and Liu, 1991). That the collagen fiber reorientation toward the circumferential direction correlated herein with the increased material stiffness suggests that remodeling is a dominant mechanism of pulmonary artery stiffening in mice in the setting of periods of hypoxia for greater than 3 weeks. Association of collagen disorientation with arterial stiffening is relatively new (Cavinato et al., 2020, 2021; Pursell and Valdez-Jasso, 2020). Increased material stiffness due to extracellular remodeling has been proposed as an underlying mechanism of disease in humans with pulmonary hypertension, thus this may have broad clinical importance (Milnor et al., 1969; Lammers et al., 2008; Westerhof et al., 2009; Saouti et al., 2010; Stevens et al., 2012; Pagnamenta et al., 2013; Dragu et al., 2015; Ghio et al., 2015; Hadinnapola et al., 2015). Increased pulmonary arterial stiffness has been shown to negatively affect right ventricular cardiac function, which is the primary predictor of survival in humans with pulmonary hypertension (Dupont et al., 2012; Stevens et al., 2012; Douwes et al., 2013; Pellegrini et al., 2014; Al-Naamani et al., 2015; Blyth et al., 2017). Increased collagen deposition has been speculated to result from chronic hypoxic exposure (Morrell et al., 1995a, b, 1997), though others have suggested that variable degradation and deposition of collagen associates with pulmonary arterial remodeling in animal hypoxia models and humans with pulmonary arterial hypertension (Zhang et al., 2020). Regardless, changes in the extracellular matrix influences changes in SMC phenotype (Safdar et al., 2014; Žaloudíková et al., 2019; Chen et al., 2020). The need to identify parameters to assess the stiffness of the pulmonary artery has been emphasized further due to the important role that stiffening of the pulmonary artery plays in diseases of the lungs such as pulmonary hypertension and chronic obstructive pulmonary disease (Sanz et al., 2008; Vivodtzev et al., 2013; Schäfer et al., 2016; Weir-McCall et al., 2018). Studies to validate the correlation of altered collagen orientation and pulmonary arterial stiffness in human tissue are underway. This study reveals further that intercellular communications between the resident cells of the pulmonary arteries play an important role in this remodeling. Further investigation is needed to better understand the underlying mechanism of the collagen disorientation and how to mitigate it under hypoxic conditions.

We confirmed that pulmonary arteries from mice have impaired contractility due to hypoxic exposure. In general, such impairment could result from dysfunctional EC signaling and/or changes in SMC phenotype (Chan and Vanhoutte, 2013). Interestingly, however, hypoxic exposure has been reported to result in EC-mediated augmentation of pulmonary and systemic arterial vasoconstriction (Chan and Vanhoutte, 2013) with sustained SMC contractility assumed to drive pulmonary vascular remodeling (Song et al., 2014). Yet, precise quantification of vasoconstriction of the pulmonary artery due to acute or chronic hypoxia has remained wanting. In the current study, we observed greater than 10% impairment of contractility of the large, elastic pulmonary artery due to chronic hypoxia. Intimal cell proliferation suggested EC proliferation in response to the hypoxic exposure, while the number of SMCs within the tunica media remained relatively unchanged. The net decreased vasocontractility despite no decrease in cell number thus suggests that there is altered function in the ECs, the SMCs, or the interaction between these cells. Further investigation is necessary to determine which of these is occurring as a result of hypoxic exposure.

Despite the increased number of intimal cells and increased expression of the endothelial-secreted vaso-constrictor peptide endothelin-1 in our study, there was no change in vasodilatory ability of the pulmonary arteries exposed to hypoxia. The decreased expression of cell adhesion genes and increased expression of Endo-MT markers suggest that ECs changed their phenotypes in response to the hypoxia (Marsboom and Rehman, 2018). There are conflicting data regarding EC proliferation due to hypoxia, some reporting EC proliferation (Fung and Liu, 1991; Porter et al., 2014), some reporting decreased ECs (Junhui et al., 2008), and some no change (Yu and Hales, 2011). It should be noted that these studies were performed with isolated ECs, separated from their natural milieu. ECs are normally juxtaposed with the innermost layer of SMCs though separated by the basement membrane and internal elastic lamina. The results herein likely reflect EC-SMC-ECM interactions that are difficult to replicate with *in vitro* experiments (Ochoa et al., 2008). Our findings may also be due to our focus on the proximal pulmonary arteries rather than more distal smaller pulmonary arteries. Regional differences of vascular biomechanics are well-described in the systemic circulation (Murtada and Humphrey, 2018). There has been an emphasis on muscular arteries and small vessels in pulmonary hemodynamics and wall mechanics, with less attention to the large elastic pulmonary artery. Previous studies have identified increased cellular density of endothelial cells in rat pulmonary arteries during the first weeks of hypoxic exposure without explanation of the cause (Fung and Liu, 1991). This study suggests that this intimal cell proliferation may reflect Endo-MT transitioning, which is supported by our observation of differentially expressed genes such as *Klf2, Fn1, Bmp6, Smad6, Tcf4*, and *Eng* (Medici and Kalluri, 2012; Chen et al., 2016). Signs of transdifferentiation also include the loss of cytoskeletal cell-cell contacts and increased transcripts for factors such as *Icam2, Gja5, Cdh11*, and *Cldn5* (Frid et al., 2002). Epigenetic changes are largely involved with Endo-MT; therefore, RNA expression may not be the optimal measure of Endo-MT. In ECs, TGF-Smad signaling pathways help bring about Endo-MT (Kovacic et al., 2019). *In vivo* studies have also shown that dysregulation of BMPR2-Wnt interactions in response to serum starvation impairs proliferation of pulmonary arterial ECs lost to apoptosis (de Jesus Perez et al., 2009). Dysregulated MAPK and Wnt signaling, as observed in our study, has been associated with a pro-proliferative, anti-apoptotic EC phenotype resulting in vascular remodeling (Awad et al., 2016). MAPK pathways comprise evolutionarily conserved kinases such as extracellular signal–regulated kinase (ERK1/2), p38 MAPK, and c-Jun NH2-terminal kinase (JNK). They receive and integrate extracellular or intracellular stimuli, regulating important cellular processes including proliferation, differentiation, metabolism, migration, survival, and apoptosis (Roux and Blenis, 2004).

Our study found decreased vaso-contractility of the pulmonary artery despite little change in SMC quantity. We hypothesized that this represented a change in SMC phenotype. The combination of decreased contractility coupled with altered gene expression of cytoskeletal proteins and increased differential expression of genes associated with cytoskeletal rearrangement (*Tgfb3, Vim, Actn4, Tnnt2, Col18a1, Ecm1*, and *Smtn*) (van der Loop et al., 1996; Worth et al., 2001; Chen et al., 2016; Pedroza Albert et al., 2020) and cell signaling (*Gne, Pde10a*, and *Map3k19*) suggests a shift from contractile to synthetic SMC phenotype (Worth et al., 2001; Rensen et al., 2007; Branchetti et al., 2013; Frismantiene et al., 2018). The differentially expressed genes associated with cytoskeletal rearrangement and the observed change of SMC orientation and alignment suggest further that the medial SMCs may have changed their orientation to align better with the stress field resulting from newly deposited or realigned adventitial collagen fibers having more of a circumferential orientation. The mechanism of this non-local phenomenon remains unclear, but contraction of SMCs reduces overall wall stress by reducing stretch in the extracellular matrix, including collagen fibers, as we observed in our mechanical measurements of this study. Therefore, (re)alignment of SMCs and collagen fibers may serve as a compensatory mechanism within pulmonary arteries to optimize the reduction of wall stress resulting from greater distension. Numerous studies have demonstrated SMC proliferation following hypoxic exposure; however, the majority of these studies have focused on medium-to-small arteries within the lungs rather than the large, elastic pulmonary arteries and many were performed *in vitro* (Minamino et al., 2001; Sodhi et al., 2001; Frid et al., 2002; Chan and Vanhoutte, 2013).

A unique aspect to this study is that scRNA sequencing and connectomic analyses allowed us to observe intercellular communication among ECs, SMCs, and fibroblasts while they remain juxtaposed in their native milieu of the pulmonary artery and exposed to hypoxia. This is the first scRNA sequencing analysis and the first connectome of pulmonary arteries in mice. Our results validate previously described differential gene expression due to hypoxic exposure, including increases in HIF, Wnt, TGFβ, and BMP related genes in ECs as well as tyrosine-kinase receptors, fibroblast growth factors, and cytoskeletal protein RNA in SMCs that have been associated with hypoxic animal models and humans with chronic hypoxia due to pulmonary arterial hypertension (Chan and Vanhoutte, 2013; Lei et al., 2016; Tao et al., 2018). We hypothesize that endothelial sensing of hypoxia triggers altered collagen orientation toward the circumferential direction, thus increasing arterial wall stiffness. We suspect that SMCs alter their orientation to better align with newly deposited or realigned collagen fibers to reduce increased overall wall stress in the pulmonary artery resulting from hypoxia. We also suspect that these changes constitute positive feedback driving continued changes due to chronic hypoxic exposures. It is unclear if these changes would continue after hypoxic exposure ceases or if these changes are reversible.

This study demonstrates the potential benefit of combining mechanical testing and imaging with cell-specific differentially expressed gene mapping and applying it to the pulmonary circulation in conjunction with transcriptomic analysis. For example, our ability to use this method to associate stiffening of the pulmonary artery with PWV may prove beneficial. Increased PWV resulting from large arterial stiffening has been associated with proinflammatory responses (increased TLR2, NF-kB activation) in pulmonary arterial ECs *in vitro* and in intralobar pulmonary arterial ECs from humans with idiopathic pulmonary arterial hypertension (Tan et al., 2014). *In vitro* studies have also demonstrated that increased flow pulsatility increased signals of inflammation and pulmonary vascular EC proliferation (*VEGF, ICAM, E-selectin, MCP-1, Flt-1*) (Li et al., 2009). We observed similar increases in gene expression of transcription factors (*Nfkb1*) and EC proliferation (*Icam1, Icam2, Flt1*) in hypoxic mice. However, we also observe how these and other changes in gene expression in ECs affect the SMCs and fibroblasts in the pulmonary arterial environment. This demonstrates that our current method of mechanical testing combined with scRNA sequencing is capable of identifying underlying mechanisms associated with increased PWV in mice following hypoxic mice. Recent evidence has suggested that failures of therapies to reduce pulsatile flow associated with arterial stiffening in patients with pulmonary arterial hypertension predicts poor clinical outcomes but the mechanisms underlying this phenomenon remain unclear (Malhotra et al., 2016; Ghio et al., 2017). A functional genetics approach as presented here applied to pulmonary arterial stiffening in humans may reveal potential mechanisms of disease or therapeutic targets. Next steps in the evolution of this method will need to include systems-level approaches to discern which of the large number of differentially expressed genes and signaling pathways have the greatest impact on functional changes and whether those impacts are protective or maladaptive. Additional methods of analysis, such as proteomics, will also be necessary to bridge transcriptomics and tissue dynamics to help identify future targets for interventions to reduce deleterious changes in pulmonary arterial structure and function while promoting compensatory changes.

Our study is limited by its small sample sizes and failure to evaluate the effects of sex on these findings; the primary goal here, however, was to establish a novel workflow. We intend to apply this method in larger studies to provide the necessary statistical power in the future. Our results are also limited by a lack of protein level analysis to validate the genetic expressions we observed. We also intend to augment our gene discovery to tissue multi-disciplinary approach with protein level investigation in future studies. Finally, despite the changes in gene expression and pathways that we observed, our study is limited in its ability to differentiate which genes or pathways are compensatory and which are maladaptive. We suspect that some of the changes we observed, such as differential gene expression of SMC cytoskeletal proteins associated with alignment of SMCs with collagen fibers, are compensatory since a decrease in arterial wall stress would be favored with SMC and collagen alignment. However, detailed gene regulatory network analysis and computational modeling of molecular and pathway interactions would be necessary to correlate changes in proteins and pathways and functional consequences. It is important to recognize that our conclusions are limited by the sample size of the sequencing data in this study. Though sufficient numbers of cells were extracted and our results revealed similar differentially expressed genes as other tissues exposed to hypoxia, more detailed analyses require a larger sample size. We intend to expand our sample size and incorporate these analyses in future work.

In summary, we observed numerous functional, structural, and transcriptomic changes in the pulmonary arteries of mice due to chronic hypoxic exposure: increased material stiffness is likely due to collagen reorientation rather than excessive collagen accumulation (fibrosis); a change in SMC phenotype resulting in decreased contractility and an alignment of the SMCs with collagen fibers; EC proliferation likely representing Endo-MT; increased pulmonary arterial PWV; and a network of cell-type specific transcriptomic changes that influence these changes. The strength of using an *ex vivo* method as described here is the complexity of the cell-cell communications is maintained. EC-SMC-ECM communication is captured because these cells were juxtaposed *in situ* up until the moment that the tissue was excised and the cells dissociated. Cellular functional and structural analyses were performed with *in situ* conditions to allow the same communications to maximize genotype:phenotype correlation. We believe that this method demonstrates the power of functional genetics which elucidates causative molecular pathways and potential genotype:phenotype correlations to justify further exploration (Maron, 2020). This multi-dimensional approach (structure, function, transcriptomics) to assessing the pulmonary artery sheds insight into its normal properties and changes due to insult. It holds great potential to investigate the pulmonary arteries in health and disease and identify potential therapeutic pathways.

## Data Availability Statement

The datasets presented in this study can be found in the GEO repository under accession number GSE182564.

## Ethics Statement

The animal study was reviewed and approved by Yale Institutional Animal Care and Use Committee.

## Author Contributions

EM performed the experiments and analyses and wrote the manuscript. AR performed the mechanical measurements and analyses and edited the manuscript. JS performed the single cell experiments and analyses and edited manuscript. CCa performed the 2-photon imaging and analyses and edited the manuscript. MR performed the differential connectome analysis and edited the manuscript. TB performed histologic experiments and analyses. CCo analyzed the single cell data. IS and GT edited the manuscript. NK supervised the single cell experiments and analyses and edited manuscript. JH supervised the biomechanical and imaging experiments and analyses and edited manuscript. All authors contributed to the article and approved the submitted version.

## Conflict of Interest

The authors declare that the research was conducted in the absence of any commercial or financial relationships that could be construed as a potential conflict of interest.

## Publisher’s Note

All claims expressed in this article are solely those of the authors and do not necessarily represent those of their affiliated organizations, or those of the publisher, the editors and the reviewers. Any product that may be evaluated in this article, or claim that may be made by its manufacturer, is not guaranteed or endorsed by the publisher.

## Supplementary Material

The Supplementary Material for this article can be found online at: https://www.frontiersin.org/articles/10.3389/fphys.2021.726253/full#supplementary-material; 10.6084/m9.figshare.16451466
