# Supplementary material for: Mechanisms of Hypoxia-Induced Pulmonary Arterial Stiffening in Mice Revealed by a Functional Genetics Assay of Structural, Functional, and Transcriptomic Data
Source: Front Physiol. 2021 Sep 14;12:726253. doi: 10.3389/fphys.2021.726253 (PMC8478173; doi:10.3389/fphys.2021.726253)
Supplement: Supplementary file 1 [file Data_Sheet_1.docx]

Supplementary Material

# Supplementary Data

The datasets presented in this study can be found in online repositories. The name of the repository and accession number are: GEO GSE182564

# Supplementary Figures

**Supplementary Figure 1.**  Pulmonary arterial wall thickness determined by 2-photon imaging of normoxic (n=2) and hypoxic (n=2) vessels at intraluminal pressures of 5, 15, 25, and 40 mmHg. There is no difference in thickness of normoxic and hypoxic vessels similar to wall thickness values calculated from mechanical measurements, as shown in Figure 2.

**
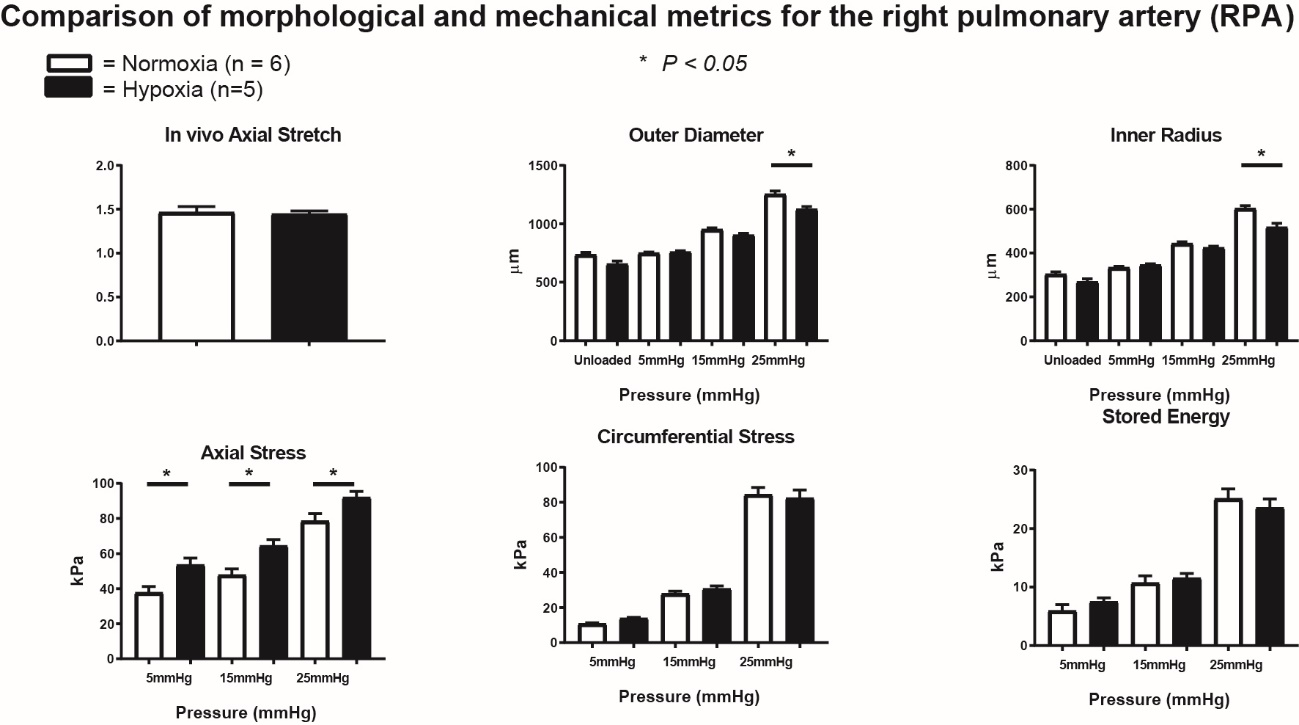
**

**Supplementary Figure 2.** Data collected (in vivo axial stretch, arterial outer diameter) or calculated (stress, stored energy) from cannulated normoxic (n=6) and hypoxic (n=5) right pulmonary arteries of C57BL6J mice using a computer-controlled testing device. Loaded (5, 15, 25 mmHg) wall inner radius and stress were calculated based on mechanical measurements. There is a decrease in outer and inner radii of hypoxic vessels compared to normoxic vessels with significant difference at the highest intraluminal pressure measured, 25 mmHg. The axial stress of hypoxic vessels is significantly increased at all pressures (5, 15, and 25 mmHg) compared to normoxic vessels. The circumferential stress of hypoxic and normoxic pulmonary arteries are similar at lower pressures; however, at 25 mmHg, the circumferential wall stress in hypoxic pulmonary arteries tend to decrease. The stored energy for hypoxic vessels is greater than normoxic vessels at diastolic pressure (5 mmHg) but less than normoxic vessels at systolic pressure (25 mmHg). * *p* < 0.05

**
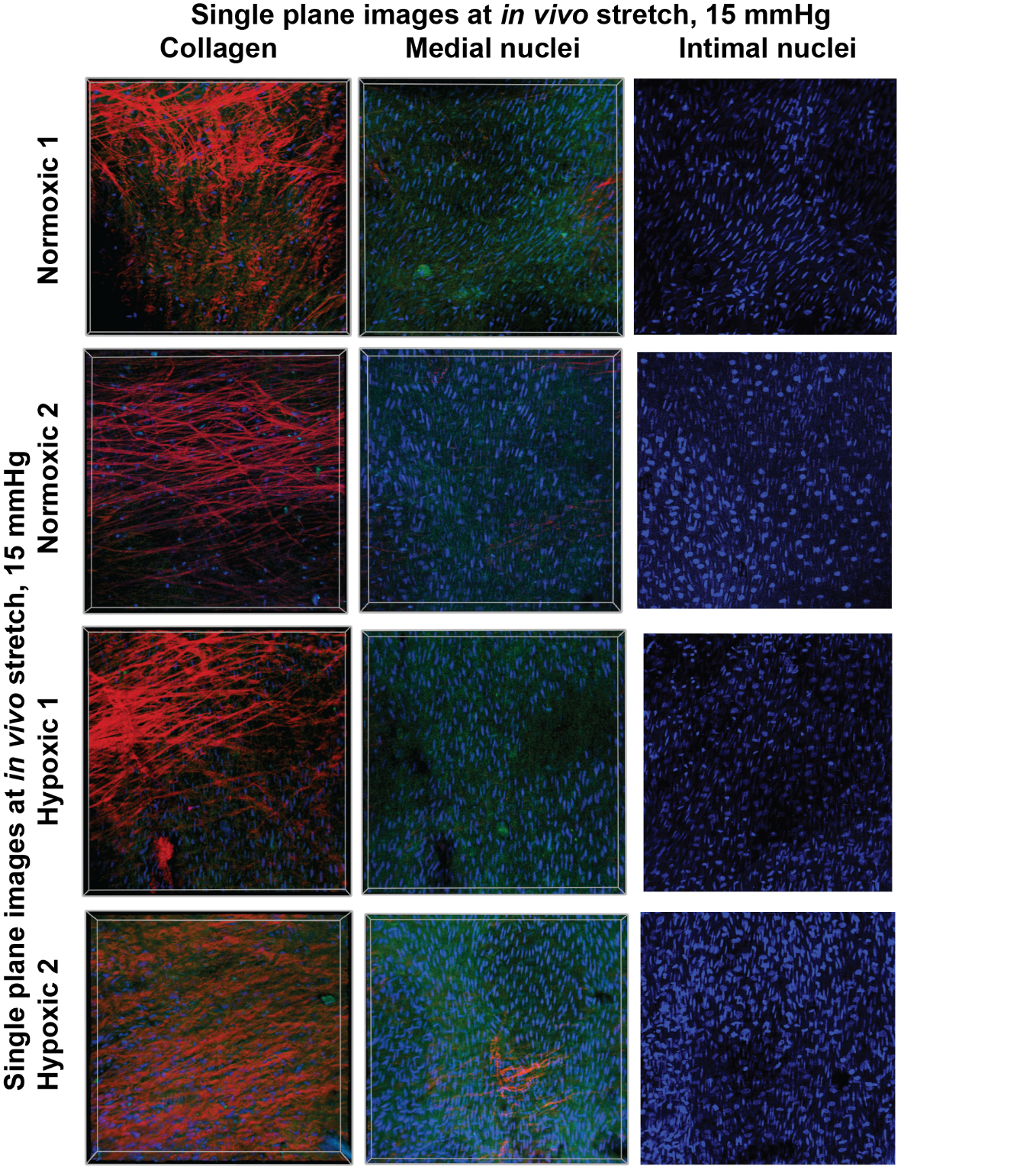
**

**Supplementary Figure 3.** Representative 2-photon images from two normoxic (n=4 total) and two hypoxic (n=4 total) samples emphasizing collagen fibers in the adventitial (red) and nuclei (blue) within both the media and intima. Red = collagen. Green = elastin. Blue = nuclei.

**Supplementary Figure 4**. Quantification of immunohistochemistry of extracellular matrix proteins. Five sections of paraffin-embedded samples from normoxic and hypoxic pulmonary arteries were analyzed to determine quantities of collagen 1 (Abcam ab34710), collagen 3 alpha 1 (Novus NB600-594), and fibronectin (Santa Cruz 69682). The trends found in hypoxic tissue (decrease in collagen 1, increase collagen 3, and decrease of fibronectin) correspond to the differential expression of these genes in hypoxic fibroblasts. These results appears consistent with degradation of existing adventitial collagen and proteins and deposition of new collagen.

**
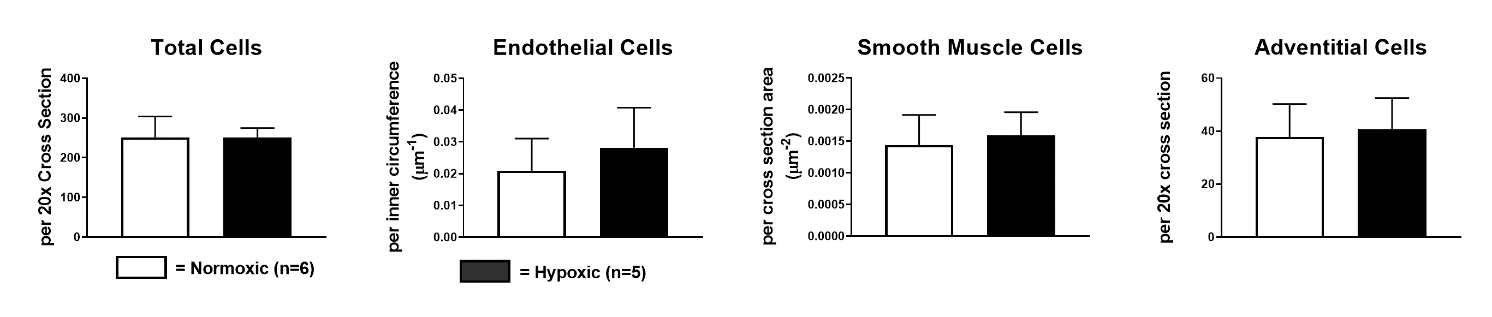

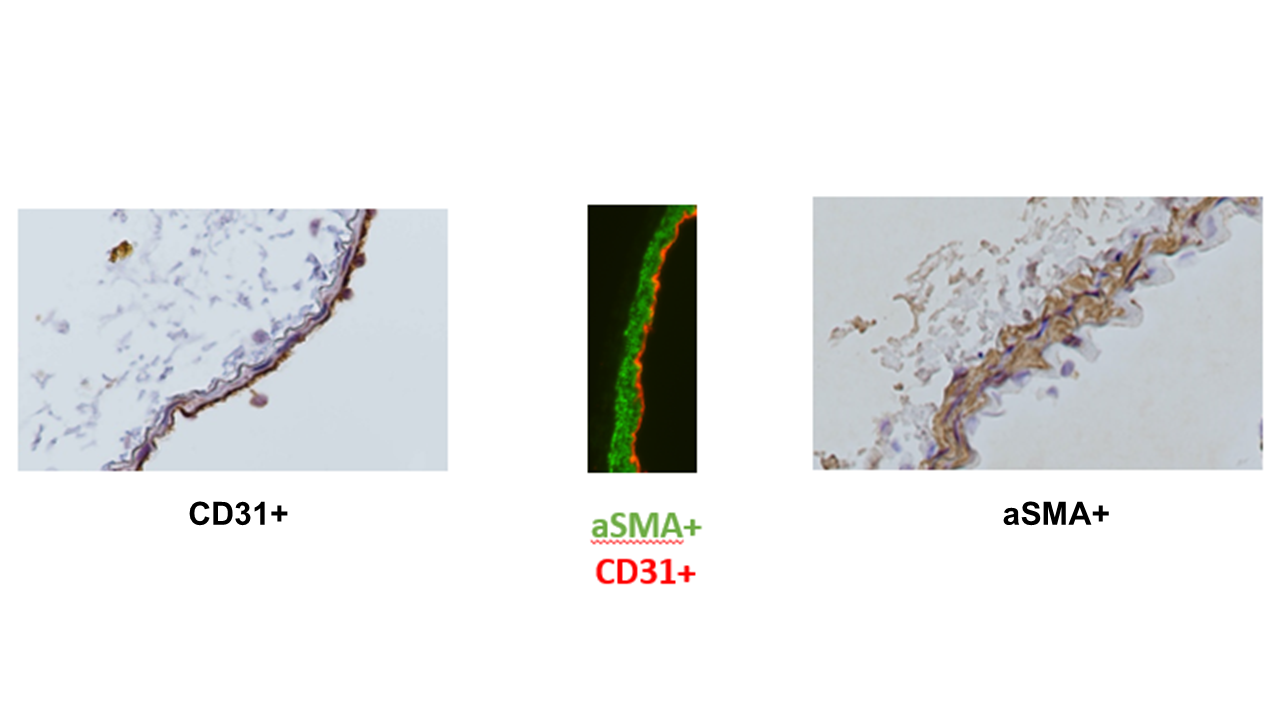
**

**Supplementary Figure 5.** H&E-stained sections provided further confirmation of changes in layer-specific cell number Five slides of each pulmonary artery were analyzed and values were averaged for each group with standard error from the mean (normoxic n=6, hypoxic n=5). The cell count was normalized based on circumference (in the case of endothelial cells) or cross-sectional area. Shown, too, are representative immuno-stained sections identifying luminal ECs (CD31+) and medial smooth muscle cells (aSMA+), noting the general lack of staining of aSMA in the adventitia, suggestive of a lack of myofibroblasts.

**Supplementary Figure 6.**  The media to adventitia thickness ratio was calculated using 2-photon imaging at intraluminal pressure of 15 mmHg. The last elastin layer furthest from the lumen of the pulmonary artery was used as the border between the media and adventitia, revealing a decreased trend in medial:adventitial collagen ratio in hypoxic samples (black, n=2) compared to normoxic samples (white, n=2) similar to findings using picrosirius red staining shown in Figure 3.

**
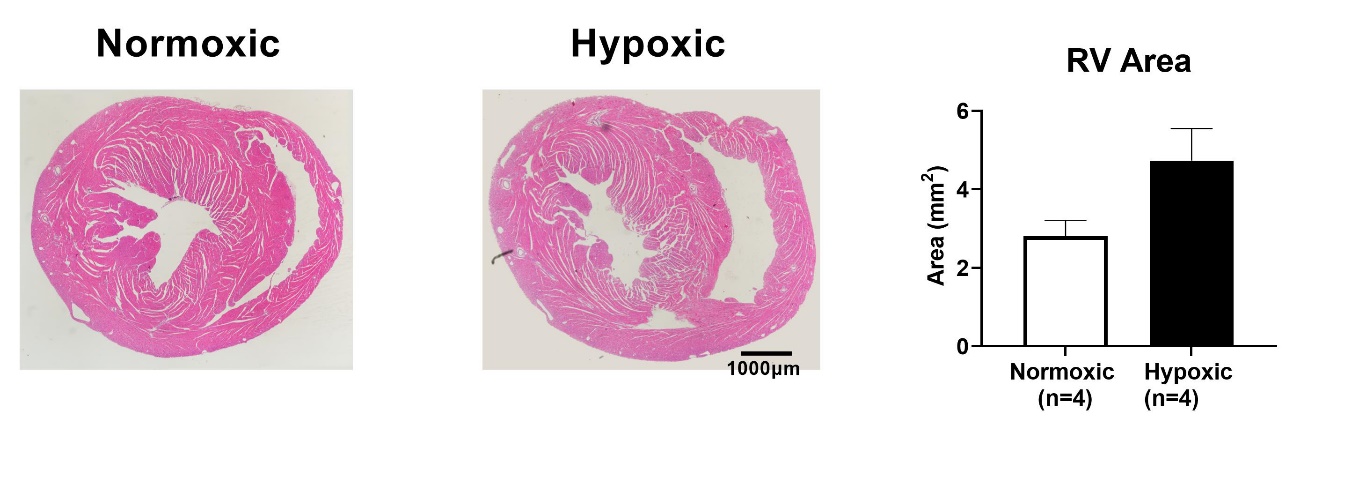
**

**Supplementary Figure 7.** Representative images of right ventricular pressure tracings, obtained using a 1 French Millar catheter, are shown from a normoxic mouse (blue) and hypoxic mouse (red) after 4 weeks of hypoxic exposure (FiO2 10%). The peak right ventricular systolic pressure (RVSP) of hypoxic mice are significantly greater than those of normoxic mice (23 ± 1.8 mmHg vs. 15 ± 0.66 mmHg, *p* < 0.05). A representative parasternal short axis cross-section of the heart of normoxic (left) and hypoxic (right) mouse are shown using paraffin embedded samples and H&E staining. Hypoxic samples revealed a significantly increased RV cavity size in hypoxic mice compared with normoxic mice (4.7 mm^2^ ± 0.8 mm^2^ hypoxic compared to 2.8 mm^2^ ± 0.8 mm^2^, *p* = 0.08). These data suggest that there are higher pressures in the right ventricle of the heart due to chronic hypoxic exposures and that right ventricular remodeling has occurred. Comparisons were made using *t*-test.

# Supplementary Tables

**Supplementary Table 1.** Material properties of normoxic (n=6) and hypoxic (n=5) pulmonary arteries using the four-fiber constitutive relation with associated best-fit material parameters.

|  |  |  |  |  |  |  |  |  |
| --- | --- | --- | --- | --- | --- | --- | --- | --- |
|  | **Material Properties** | | | | | | |  |
|  |  | **Normoxia, C57BL6/J** | | | **Hypoxia, C57BL6/J** | | |  |
|  |  | **n** | **=** | **6** | **n** | **=** | **5** |  |
|  | **Elastic Fibers** |  |  |  |  |  |  |  |
|  | *c* (kPa) | 9.33 | ± | 0.89 | 2.73 | ± | 1.38 |  |
|  |  |  |  |  |  |  |  |  |
|  | **Axial Collagen** |  |  |  |  |  |  |  |
|  | *c*_1_^1^ (kPa) | 2.46 | ± | 0.55 | 2.91 | ± | 1.98 |  |
|  | *c*_2_^1^ | 2.57 | ± | 0.80 | 2.41 | ± | 1.11 |  |
|  |  |  |  |  |  |  |  |  |
|  | **Circumferential Collagen and Smooth Muscle Cell** |  |  |  |  |  |  |  |
|  | *c*_1_^2^ (kPa) | 1.23 | ± | 0.33 | 4.49 | ± | 0.56 |  |
|  | *c*_2_^2^ | 0.37 | ± | 0.13 | 0.0020 | ± | 0.0007 |  |
|  |  |  |  |  |  |  |  |  |
|  | **Diagonal Collagen** |  |  |  |  |  |  |  |
|  | *c*_1_^3,4^ (kPa) | 6.83 | ± | 1.75 | 10.08 | ± | 1.61 |  |
|  | *c*_2_^3,4^ | 0.33 | ± | 0.03 | 0.32 | ± | 0.05 |  |
|  | *α_o_* (deg) | 35.55 | ± | 1.80 | 28.45 | ± | 2.00 |  |
|  | RMSE | 0.10 | ± | 0.01 | 0.08 | ± | 0.01 |  |
|  |  |  |  |  |  |  |  |  |
|  |  |  |  |  |  |  |  |  |
|  |  |  |  |  |  |  |  |  |
